# The Fully Oxidized State of the Glutamate Coordinated O_2_-Tolerant [NiFe]-Hydrogenase Shows a Ni(III)/Fe(III) Open-Shell Singlet Ground State

**DOI:** 10.1021/jacs.3c02438

**Published:** 2023-05-09

**Authors:** Ravi Kumar, Matthias Stein

**Affiliations:** † Max Planck Institute for Dynamics of Complex Technical Systems, Molecular Simulations and Design Group, Sandtorstrasse 1, 39106 Magdeburg, Germany

## Abstract

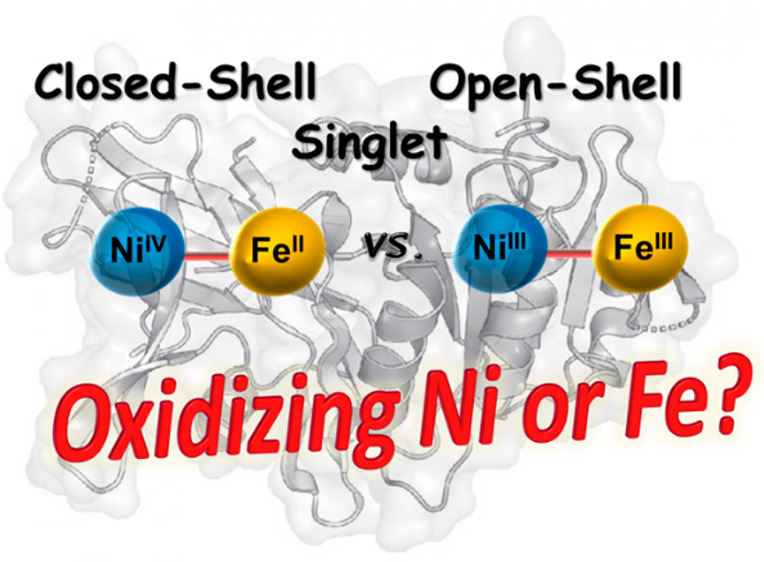

The oxygen tolerance of the [NiFe]-hydrogenase from *H. thermoluteolus* was recently assigned to originate from an unusual coordination sphere of the active site nickel atom (Shomura et al. *Science***2017**, *357*, 928–932, 10.1126/science.aan4497). In the oxidized state, a terminal cysteine residue is displaced by a bidentate coordinating nearby Glu32 and thus moves to occupy a third μ-cysteine bridging position. Spectral features of the oxidized state were assigned to originate from a closed-shell Ni(IV)/Fe(II) state (Kulka-Peschke et al. *J. Am. Chem. Soc.***2022**, *144*, 17022–17032, 10.1021/jacs.2c06400). Such a high-valent nickel oxidation state is unprecedented in biological systems. The spectral properties and the coordination sphere of that [NiFe]-hydrogenase can, however, also be rationalized by an energetically lower broken-symmetry Ni(III)/Fe(III) state of the active site which was not considered. In this open-shell singlet, the ligand-mediated antiferromagnetic spin-coupling leads to an overall *S* = 0 spin state with evenly distributed spin densities over the metal atoms. Experiments are suggested that may clarify the final assignment of redox states.

In this Communication, we provide first scientific arguments why we consider the assignment of the unprecedented Ni(IV) oxidation state in the soluble hydrogenase (SH) as ambiguous and not fully plausible.^1^ Hydrogenases are a group of metalloenzymes that catalyze the conversion of dihydrogen into protons and electrons and the reverse reaction. According to their active site compositions they are classified as [FeFe]-, [NiFe]-, and [Fe]-hydrogenases.^2,3^
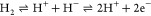
1

The [NiFe]-hydrogenases tend to be biased toward H_2_ oxidation, and the [FeFe]-hydrogenases toward the production of molecular hydrogen.^4^

The group I [NiFe]-hydrogenases, such as *Desulfovibrio (D.) vulgaris* Miyazaki F and *D. gigas*, shuttle between diamagnetic Ni(II) and paramagnetic Ni(III) oxidation states during hydrogen turnover with an electron flow from a chain of iron–sulfur clusters. Ni-A (“unready”) and Ni-B (“ready”) are the fully oxidized, paramagnetic states with an S = 1/2 ground state, both with low-spin Ni(III)/Fe(II) cores.^5^ Several crystal structures of the “as-isolated” enzymes are available and reveal the presence of an oxygenic ligand.^6−8^ The “reduced” Ni-C state^9,10^ is a doublet Ni(III)/Fe(II) core with a μ-bridging hydride.^11,12^ Ni-L is in a Ni(I) oxidation state which can either be a photoreduced and only stable at low temperature species;^13^ for other strains, however, it is also detectable in the dark at ambient temperature which might suggest an involvement in the catalytic cycle.^14^ There are no large structural rearrangements regarding the active site: Ni···Fe distances, and Ni–SCys bonds only show very minor changes during H_2_ turnover.^4^ Crystal structures of the reduced forms show the removal of the bridging ligand that is present in the oxidized states (OH^–^ or OOH^–^).^10^ In all crystal structures, the nickel atom is coordinated by two terminal and two bridging cysteine residues. The “fully reduced” Ni-R state is EPR-silent. Its structure was solved at a subatomic resolution of 0.89 Å that tentatively allowed the assignment of the positions of a hydride (μ-bridging) and a protonated terminal cysteine (see Figure 1).^15^

**Figure 1:**
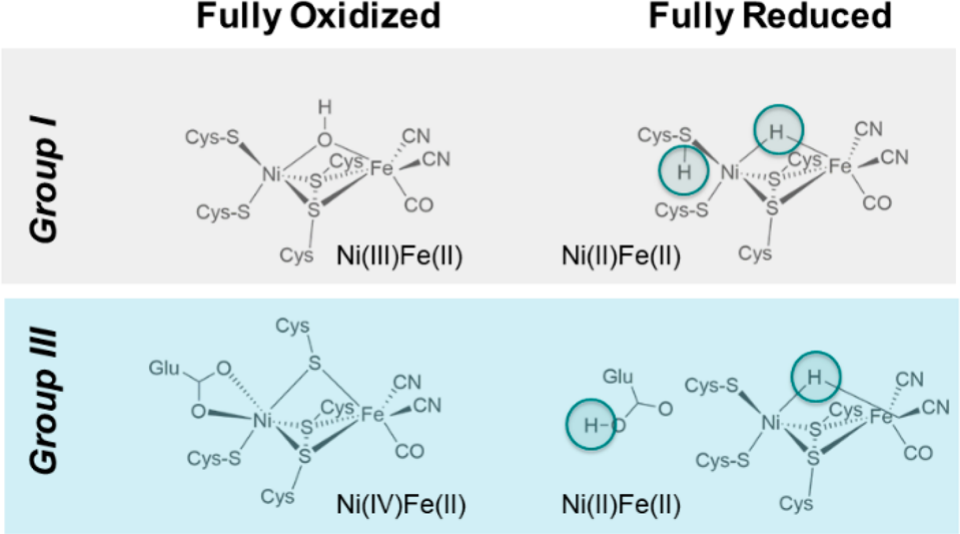
Structural details of the active sites of [NiFe]-hydrogenases in fully oxidized and reduced states. Top: group I, such as *D. vulgaris* Miyazaki F and *D. gigas*. Bottom: group III, e.g. soluble hydrogenase *H. thermoluteolus* or *R. eutropha*.

In [NiFe]-hydrogenases, the active site iron atom is coordinated by biologically uncommon strong inorganic ligands (CO and CN^–^). They were first detected by FTIR spectroscopy and are able to give state-specific information about the binding situation during the catalytic cycle.^16−18^ The absence of electron–nuclear spin hyperfine interactions in Q-band ENDOR^19^ and ^57^Fe Mössbauer^20,21^ enabled the assignment of a mixed-valence low-spin 3d^7^ Ni(III) (*S* = 1/2) and low-spin 3d^6^ Fe(II) (*S* = 0) species for oxidized Ni-A, Ni-B, and reduced Ni-C.

Some [NiFe]-hydrogenase enzymes show improved tolerance toward the presence of oxygen, enabling H_2_ oxidation under aerobic conditions. Critical factors to avoid oxygenation of the active site may, for example, be an unusual [4Fe-3S](Cys)_6_ cluster in proximity to the active site.^22−24^ Further work to elucidate the oxygen-tolerance of some [NiFe]-hydrogenases has also identified a narrow and hydrophobic access channel to the active site,^25−27^ the presence of a selenocysteine,^9,28^ alterations in the coordination sphere of the proximal [FeS]-cluster by two additional cysteine residues,^22,23,29^ and an amide nitrogen and glutamate coordination.^30^ There are no conformational changes at or in the vicinity of the NiFe active site that are firmly assigned to be functionally responsible for the oxygen tolerance.

Crystal structures of the oxygen-tolerant group III NAD^+^-reducing [NiFe]-hydrogenase from *Hydrogenophilus thermoluteolus* (*Ht*SH) are available in the air-oxidized (PDB: 5XF9) and reduced (PDB: 5XFA) states.^31^ The oxidized active site shows an unusual distorted octahedral six-coordinate nickel with three bridging cysteines, one terminal cysteine, and a bidentate Glu32 coordination (see Figure 1). The iron site is coordinated by two cyanide and one carbon monoxide ligand. The IR spectrum of the oxidized state features an unusual CO vibration band at 1993 cm^–1^ that is distinct from all other [NiFe]-hydrogenases.^32^ The X-ray structure of the reduced state, its vibrational and EPR signatures, in contrast, are similar to those of typical reduced group I [NiFe]-hydrogenase enzymes and allowed the tentative assignment of a μ-bridging hydride and possibly a protonated Glu32 in the fully reduced form (see Figure 1).^31,32^

Recently, the effect of such a reversible glutamate coordination was investigated in order to link crystallographic and spectral features.^1^ Ultrafast and two-dimensional infrared spectroscopy were used to give details into the structure and dynamics of the formation of the conformationally strained oxidized structure. The analysis and interpretation of experimental data was supported by density functional theory (DFT) calculations.

Based on comparison with spectral data, the reduced state was assigned to a Ni-C-like “model 7” which is identical to that of group I hydrogenases (see X-ray structure 5XFA at 2.70 Å resolution). For the fully oxidized state of the enzyme, however, a formal Ni(IV)/Fe(II) state with a terminally coordinated glutamate and three bridging cysteine residues (t-Glu/Ni(IV)(μ-Cys)_3_Fe(II)(CN)(CO)_2_) (“model 20”) was suggested (see Figure 1, 5XF9 at 2.58 Å resolution). The terminal Glu32 bidentate coordination would displace one of the terminal cysteines into a bridging position between the nickel and iron atoms. This biologically unprecedented oxidation state would be a closed-shell singlet of a low-spin Ni(IV, *S* = 0) and a low-spin Fe(II, *S* = 0) center, both in an octahedral coordination environment. We could reproduce the computational results from ref (1) (structural parameters, *g*-values, and IR spectra, see Supporting Information (SI)) by using identical methods and cluster models. We agree with the authors’ plausible models for Ni_r_–S and Ni(III)_r_–t-OH. However, the assignment of a Ni(IV) state in their “model 20” for the fully oxidized state of the active site is not unambiguous. In the triply thiolate-bridged Ni–Fe center, different formal oxidation and spin states are feasible and give spectral features in agreement with experiment.

Figure 2 shows the experimental and calculated IR spectra of the most plausible “model 20” of the fully oxidized from ref (1) and from our work.

**Figure 2:**
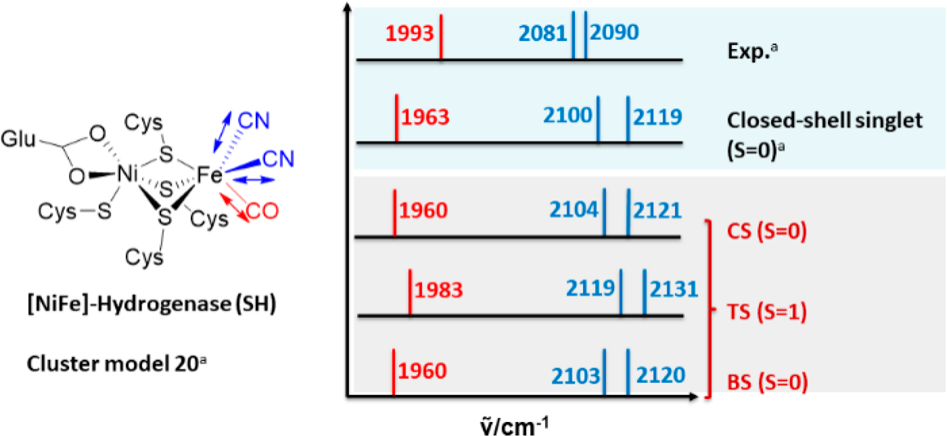
Comparison of experimental and calculated vibrational spectra for the oxidized state of the [NiFe]-hydrogenase *HtSH*. ^a^ Experimental and Ni(IV)/Fe(II) calculated spectra are taken from ref (1). Closed-shell (CS), triplet state (TS), and broken-symmetry (BS) spectra are from this work.

The calculated IR spectra for the oxidized state of *HtSH* are compared to experimental data. For the closed-shell singlet Ni(IV) Fe(II), our calculations reproduce the ones from ref (1) very well to within ∼3 cm^–1^, given the use of a different code with different integration schemes, the use of a different (6-31G(d) basis set for nonmetal atoms and the fixing of Cα atoms in ref (1). This shows that the closed-shell (*S* = 0, “CS” in our notation) state is one possible model for the oxidized state (see Figure 2). However, there is another electronic state that gives an IR spectrum almost identical to the closed-shell solution and experiment. The “closed-shell” system consists of a formal Ni(IV) 3d^6^ and an Fe(II) 3d^6^. In the broken-symmetry (“BS”) state, a Ni(III) 3d^7^ (*S* = 1/2) is antiferromagnetically coupled to an Fe(III) 3d^5^ (*S* = 1/2) to give an “open-shell” singlet (see Figure 3). The IR spectrum suggests that the BS solution is thus equally possible and a further candidate model for oxidized *HtSH*. The triplet state results are deviating more from experiment, in particular for the CN^–^ vibrations.

**Figure 3:**
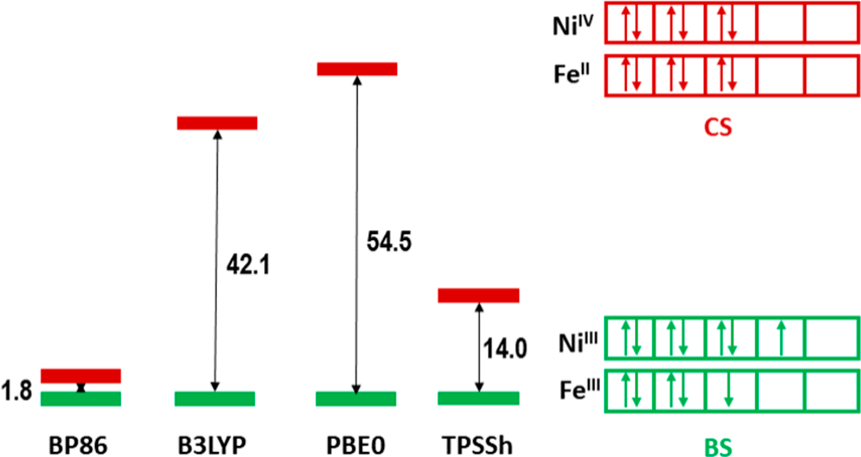
Calculation of the stabilization energy in kJ mol^–1^ of the spin-coupled broken-symmetry (BS; Ni 3d^7^/Fe 3d^5^ Ni(III)/Fe(III)) vs the closed-shell (CS; Ni 3d^6^/Fe 3d^6^ Ni(IV)/Fe(II)) electronic configurations for the oxidized state of *HtSH*.

The broken-symmetry solution is obtained from the ferromagnetic high-spin state,^33,34^ and the energy splitting between the states is calculated using the approximate spin projection formula^35^
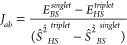
2

Figure 3 shows the energy splitting between the closed-shell and broken-symmetry states for a number of exchange-correlation functionals such as the GGA BP86,^36−38^ hybrid functionals B3LYP^39^ and PBE0,^40−42^ and TPSSh.^43^ More results can be found in the Supporting Information.

All calculations consistently report the broken-symmetry to be lower in energy than the closed-shell solution. Even for the GGA BP86, which is known to overstabilize low-spin states, the BS state is slightly lower in energy than the closed-shell state. The hybrid functionals B3LYP (with 20% HF exchange) and PBE0 (25% HF exchange) give larger energy splittings of 42 and 55 kJ mol^–1^, respectively. In the SI, the effect of systematic variations of the amount of exact exchange on Δ*E*_HS-BS_ can be seen. The meta-hybrid GGA TPSSh functional was shown to give the most reliable exchange coupling constants in comparison with experiment^44^ and was also performing superior to double-hybrid density functionals.^45^ Here, the calculated stabilization energy is 14 kJ mol^–1^.

Figure 4 shows one example for a BS-DFT (*S* = 0) model for the oxidized state. Structural parameters of the BS-DFT are in good agreement with experiment (see SI). The unpaired spin density is equally distributed over the nickel (α-spin ↑, 0.81) and iron (β-spin ↓, −0.82) atoms. The inverse coupling of Ni(III, ↓) with Fe(III, ↑) is equally feasible and isoenergetic. High-valent Ni(IV) oxidation states are not reported for enzymatic systems so far but are known for highly active catalysts and may be catalytic intermediates which are stabilized by basic or chelating ligands.^46−48^

**Figure 4:**
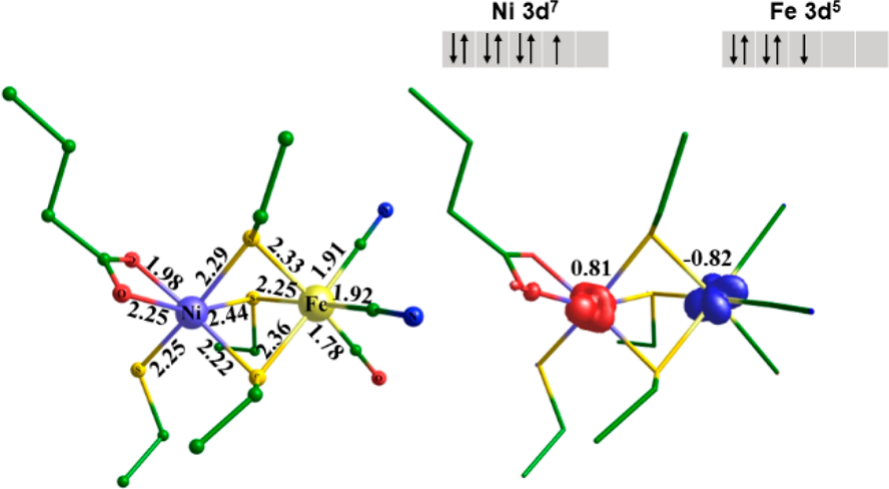
Antiferromagnetic coupling between low-spin Ni(III) (3d^7^, ↑) and low-spin Fe(III) (3d^5^, ↓) in a model of the fully oxidized state from *HtSH*. Left: structural parameters in Å (TPSSh/def2-TZVP). Right: unpaired spin density distribution (isocontour value of 0.03 au).

Biomimetic nickel–iron complexes^49−52^ have shown that formal oxidation states and electron spin distributions in mixed-valence (NiFe)^3+^ can be controlled by the nature of terminal ligands. Even the iron atom in a strong ligand field can be partially redox active. ^57^Fe Mössbauer studies on [NiFe]-hydrogenases, for example in refs (53 and 54), however, are consistent with a Fe(II) low-spin state. Nickel and iron atoms in the enzyme and heterobiometallic complexes are usually tetra- or penta-coordinate with two bridging thiolate ligands.

In an octahedral coordination of nickel and iron, as suggested for the fully oxidized *HtSH*_*ox*_, an electronic exchange coupling between the nickel and iron spin is possible. Antiferromagnetic coupling between low-spin Ni(III, ↓) and low-spin Fe(III, ↑) in triply thiolate-bridged complexes^55^ gives rise to characteristic ^57^Fe Mössbauer parameters (δ = 0.26 mm s^–1^; |Δ*E*_Q_| = 1.85 mm s^–1^). Also the antiferromagnetic exchange between octahedral thiolate-bound high-spin Ni(II, *S* = 1) with octahedral low-spin Fe(III) gives similar values (δ = 0.32 mm s^–1^; |Δ*E*_Q_| = 1.83 mm s^–1^).^56^

An unambigous discrimination between the possible closed-shell and open-shell singlet configurations is very challenging. Structural parameters are indistiguishable (see Table S1) and so are IR spectra (see Figure 2). Possibly, ^57^Fe Mössbauer studies on the oxidized state of *HtSH* might be be able to resolve the spin density at the iron nucleus and thus its oxidation state. Table 1 gives the calculated Mössbauer parameters for the closed-shell and broken-symmetry electronic configurations.^57^

**Table 1:** Calculated ^57^Fe Mössbauer Parameters for Oxidized *HtSH*

Fully oxidized state of SH	δ/mm s^–1^	|Δ*E*_Q_|/mm s^–1^
Closed-shell	0.18	0.49
Broken-symmetry	0.12	2.53

The isomer shift δ is proportional to the electron density at the nucleus which is varying due to different d-orbital shieldings.^58^ For the *HtSH*, expected differences in isomer shifts δ are small. The quadrupole splitting Δ*E*_Q_ provides information about the local charge asymmetry at the iron site.^59^ The quadrupole splitting of the broken-symmetry state is in good agreement with experiments on the Ni(II)/Fe(III) model complex.^56^ The intramolecular exchange interaction between octahedral low-spin Fe(III) (*S* = 1/2) and Ni(II) (*S* = 1) is mediated by the three μ-bridging thiolate ligands, which are also present in the model for oxidized *HtSH*. Thus, ^57^Fe Mössbauer might be able to resolve the formal oxidation state of the iron in fully oxidized *HtSH*.

The issue whether the oxidized state of *HtSH* is a closed-shell Ni(IV)/Fe(II) or a spin-coupled Ni(III)/Fe(III) is not only of relevance for an understanding of the oxygen tolerance of group III [NiFe]-hydrogenases. It is also important with regard to the general accessibility of a Ni(IV) oxidation state in biological systems. It has to be considered that the description of the oxidation states by a localized model may be oversimplified. However, the resonance structures involving Ni(IV)/Fe(II) will not have the same weighting as the broken-symmetry Ni(III)/Fe(III) solution.

**Computational Details.** All calculations were performed using Turbomole 7.5.1^60^ using the given exchange-correlation functionals and Ahlrichs’-type basis sets in the SI. Mössbauer parameters and variation of HF exchange (in TPSS, TPSSh, and TPSS0) were performed using ORCA^61−63^ (see Supporting Information for more details).

## Supporting Information Available

Additional computational details, structural data, energies, spectroscopic results and Cartesian coordinates (PDF)
