# Supplementary material for: The Fully Oxidized State of the Glutamate Coordinated O2-Tolerant [NiFe]-Hydrogenase Shows a Ni(III)/Fe(III) Open-Shell Singlet Ground State
Source: J Am Chem Soc. 2023 May 9;145(20):10954–9. doi: 10.1021/jacs.3c02438 (PMC10214449; doi:10.1021/jacs.3c02438)
Supplement: Supplementary file 1 — ja3c02438_si_001.pdf [file ja3c02438_si_001.pdf]

Supporting Information

The Fully Oxidized State of the Glutamate Coordinated O_2-Tolerant [NiFe]-Hydrogenase Shows a Ni(III)/Fe(III) Open-Shell Singlet Ground State

Ravi Kumar[a], Matthias Stein[a,*]

[a]Max Planck Institute for Dynamics of Complex Technical Systems, Molecular Simulations and Design Group, Sandtorstrasse 1, 39106 Magdeburg, Germany.

*Corresponding author: matthias.stein@mpi-magdeburg.mpg.de

Computational Details

All the geometry optimizations have been performed using TURBOMOLE rev. V7.5.1.[1-2] The calculations were performed using density functional theory (DFT) [3] with dispersion correction[4] and Becke–Johnson damping (D3(BJ)). [5] Various functionals such as BP86,[6-8] B3LYP,[9-10] PBE0,[11-12] and TPSSh[13-14] were evaluated in order to obtain consistent results across different families of functional. The basis set used here is def2-TZVP for all the atoms.[15] Structures were optimized for both the closed-shell and broken-symmetry states. The second derivatives were calculated for both solutions to obtain vibrational frequencies and to identify them as minima. All broken-symmetry states are obtained from the high-spin triplet states using broken symmetry (BS) approach proposed by Noodleman and co-workers and implemented in the TURBOMOLE package.[16-19] High-spin states for both the metal centers (Ni, Fe) have been first optimized and then a spin-flip is performed for one of the centers (Ni or Fe) to obtain the broken symmetry solutions. The coupling constants were calculated using the Yamaguchi formula (see main text). ORCA version 5.0.3 was used to calculate Mössbauer parameters and to scan the admixture of Hartree-Fock (HF) exchange in TPSS functionals (see Table Figure S1).[22-23] For B3LYP, HF exchange variation was performed in Turbomole (see Figure S2). Mössbauer isomer shifts (δ) were computed at the B3LYP(D3BJ)/CP(PPP)(Fe),def2-TZVP(all remaining atoms)(CPCM(acetonitrile)) level of theory using the RIJCOSX approximation and the derived linear equation from Römelt *et al.* ($\delta = \alpha\,(\rho - C) + \beta$) ($\alpha = -0.366, \beta = 2.852\ and\ C = 11810$).[35]

Table S1: Comparison of experimental bond lengths and bond angles from X-ray structure, the previously suggested 'model20', and our closed-shell and broken-symmetry solutions with different functionals.

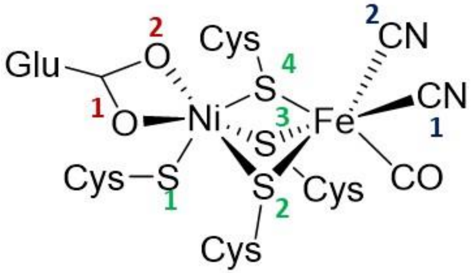

Bond lengths (Å)														
**Method**	**Species**	**Ni-Fe**	**Ni-S1**	**Ni-S2**	**Ni-S3**	**Ni-S4**	**Ni-O1**	**Ni-O2**	**Fe-CN1**	**Fe-CN2**	**Fe-CO**	**Fe-S2**	**Fe-S3**	**Fe-S4**
**X-Ray[20]**		2.864	2.356	2.346	2.347	2.341	2.192	2.242	1.774	1.796	1.772	2.348	2.349	2.351
**BP86**	**model20 [21]**	3.054	2.274	2.307	2.340	2.282	2.149	2.122	1.902	1.902	1.746	2.395	2.268	2.371
	**CS**	3.003	2.250	2.193	2.259	2.312	2.152	2.066	1.888	1.898	1.740	2.299	2.264	2.358
	**BS**	3.003	2.248	2.187	2.258	2.318	2.154	2.061	1.888	1.898	1.741	2.303	2.266	2.351
**TPSSh**	**CS**	2.993	2.258	2.181	2.233	2.300	2.040	2.050	1.899	1.910	1.752	2.283	2.296	2.341
	**BS**	3.006	2.249	2.217	2.437	2.292	2.249	1.982	1.918	1.911	1.778	2.358	2.247	2.325
**B3LYP**	**CS**	3.034	2.281	2.196	2.244	2.327	2.041	2.097	1.914	1.924	1.763	2.321	2.353	2.378
	**BS**	3.023	2.271	2.254	2.502	2.325	2.296	1.999	1.933	1.928	1.807	2.388	2.303	2.330

Bond Angles (°)												
**Method**	**Species**	**Ni-S2-Fe**	**Ni-S3-Fe**	**Ni-S4-Fe**	**O1-Ni-O2**	**CN1-Fe-CN2**	**S4-Fe-CO**	**S3-Fe-CN1**	**S2-Fe-CN2**	**S2-Ni-O2**	**S3-Ni-O1**	**S4-Ni-S1**
**X-Ray[20]**		75.2	75.1	75.2	60.1	91.0	165.8	161.0	169.3	175.9	152.4	168.4
**BP86**	**model20[21]**	81.0	83.0	82.0	62.4	88.9	178.0	167.8	172.4	170.1	167.9	166.2
	**CS**	83.9	83.2	80.0	62.9	93.0	175.8	165.7	171.7	171.4	162.9	173.8
	**BS**	83.9	83.2	80.1	63.0	93.1	175.6	165.5	172.0	170.9	162.0	174.4
**TPSSh**	**CS**	84.2	82.8	80.3	64.4	93.9	174.9	165.8	171.4	170.5	165.1	173.6
	**BS**	82.1	79.7	81.2	62.3	88.9	174.2	168.5	175.0	171.8	160.5	172.1
**B3LYP**	**CS**	84.4	82.6	80.3	63.4	94.6	174.1	165.4	171.0	168.5	165.6	173.1
	**BS**	81.2	77.9	81.0	61.1	88.5	171.8	169.8	176.7	168.9	160.4	171.9

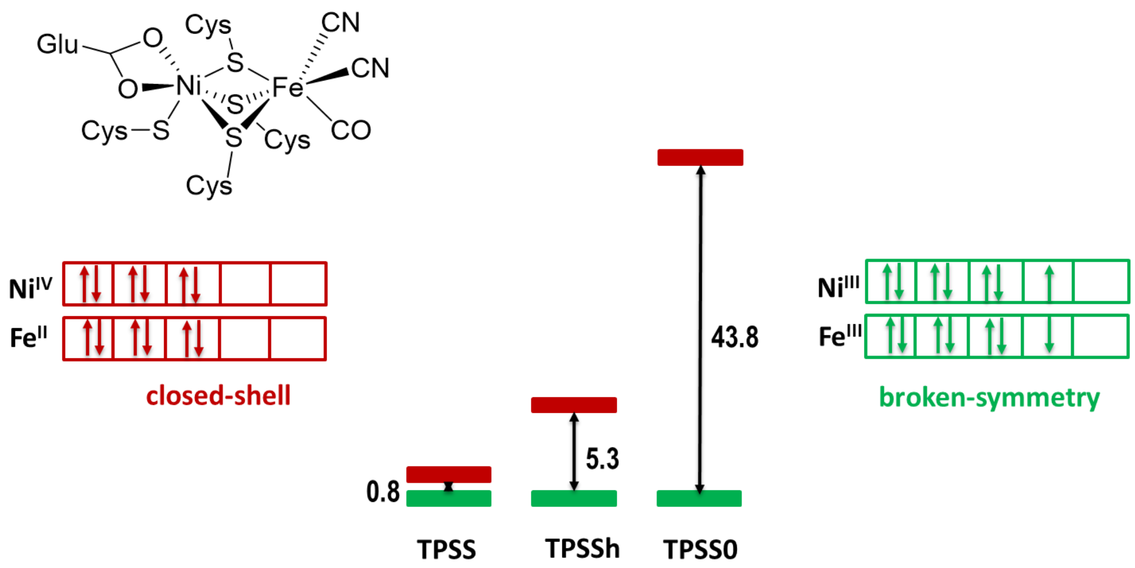

Figure S1: Energy splitting between closed-shell (CS) and broken-symmetry (BS) singlet structures using TPSS, TPSSh and TPSS0 functionals with varying degrees of HF exchange. Single-point calculations were performed on TPSSh/def2-TZVP structures with ORCA.[22-23]

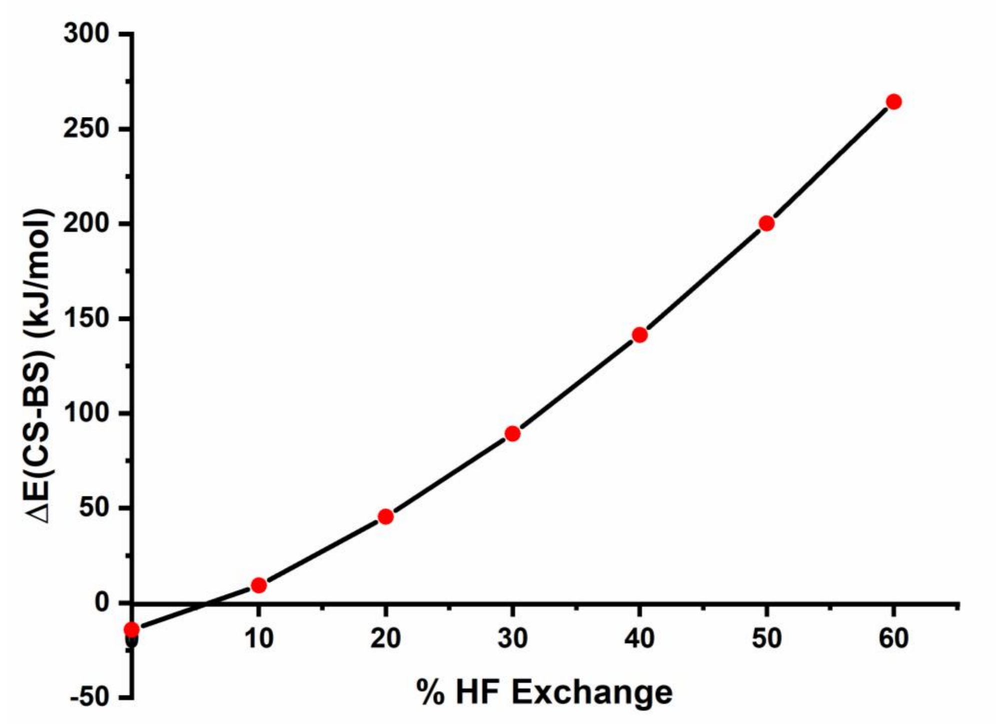

Figure S2: Dependence of spin state splitting between closed-shell (CS) and broken-symmetry (BS) states in kJ/mol on the amount HF exchange in the B3LYP.

Table S2: Comparison of BP86/def2-TZVP computed IR frequencies (cm^{-1}) of the active site from [ref. 21] and our results (in brackets).

**ν (cm^{-1})**	**ν (C=O)**	**ν (C=N)_1**	**ν (C=N)_2**	
**model1**	1882 (1860)	2048 (2051)	2078 (2071)	**t-Glu/µ-Cys (Ni^{II})**
**model7**	1882 (1866)	2053 (2048)	2068 (2063)	**t-Cys/µ-OH (Ni^{II})**
**model9**	1919 (1912)	2079 (2082)	2103 (2100)	**t-Glu/µ-Cys (Ni^{III})**
**model15**	1922 (1912)	2075 (2073)	2087 (2087)	**t-Cys/µ-OH (Ni^{III})**
**model17**	1913 (1906)	2080 (2078)	2080 (2095)	**t-OH/µ-Cys (Ni^{III})**
**model20**	1963 (1960)	2100 (2104)	2119 (2121)	**t-Glu/µ-Cys (Ni^{IV})**
**model29**	1974 (1967)	2092 (2102)	2102 (2111)	**t-Cys/µ-OH (Ni^{IV})**
**Exp.**				
**Ni(IV)$_r$-Hex (WT)**	1993	2081	2090	[36]
**Ni(IV)$_r$-Hex (E32D)**	1998	n.a.	n.a.	[21]
**Ni(III)$_r$-Hex (WT)**	1964	2087	2098	[36]
**Ni(III)$_r$-Hex (E32D)**	1969	n.a.	n.a.	[21]
**Ni(III)$_r$-Hex' (WT)**	1971	n.a.	n.a.	[21]
**Ni(III)$_r$-t-OH (E32A)**	1971	n.a.	n.a.	[21]
**Ni(III)$_r$-t-OH (E32Q)**	1974	n.a.	n.a.	[21]
n.a. = not assigned. Labelling of enzymes states according to ref. [21]				

Table S3: Comparison of experimental and computed EPR g-tensor principal values of the Ni(III)$_r$-Hex active site models from ref. [21] and our results (in brackets).

	**g_x**	**g_y**	**g_z**	
**model 09**	2.22 (2.17)	2.16 (2.14)	2.06 (2.04)	**t-Glu/µ-Cys**
**model 15**	2.22 (2.20)	2.13 (2.13)	2.06 (2.05)	**t-Cys/µ-OH**
**model 17**	2.18 (2.17)	2.16 (2.15)	2.04 (2.03)	**t-OH/µ-Cys**
**Exp.**				
**Ni(III)$_r$-Hex (WT)**	2.26	2.13	2.04	[21]
**Ni(III)$_r$-Hex' (WT)**	2.25	2.13	2.04	[21]
**Ni(III)$_r$-t-OH (E32Q)**	2.16	2.14	2.01	[21]
**Ni(III)$_r$-t-OH (E32A)**	2.17	2.15	2.01	[21]

Table S4: Energy splitting between high-spin (HS) and broken-symmetry (BS) states (in kJ/mol) and as coupling constant J (in cm^{-1}). J is calculated using the Yamaguchi formula (shown below). [24-25] For hybrid functionals, the percentage of Hartree-Fock (HF) exchange is given. The triplet state is higher in energy for all hybrid functionals.

	ΔE(HS-BS) (kJ/mol)	J (cm^{-1})	% HF
BP86	-14.9	-614	0.0
B3LYP	0.3	21	20.0
PBE0	1.2	102	25.0
TPSSh	0.4	31	10.0

$$J = \frac{E(BS) - E(HS)}{< S^2 >_{HS} - < S^2 >_{BS}}$$

Yamaguchi

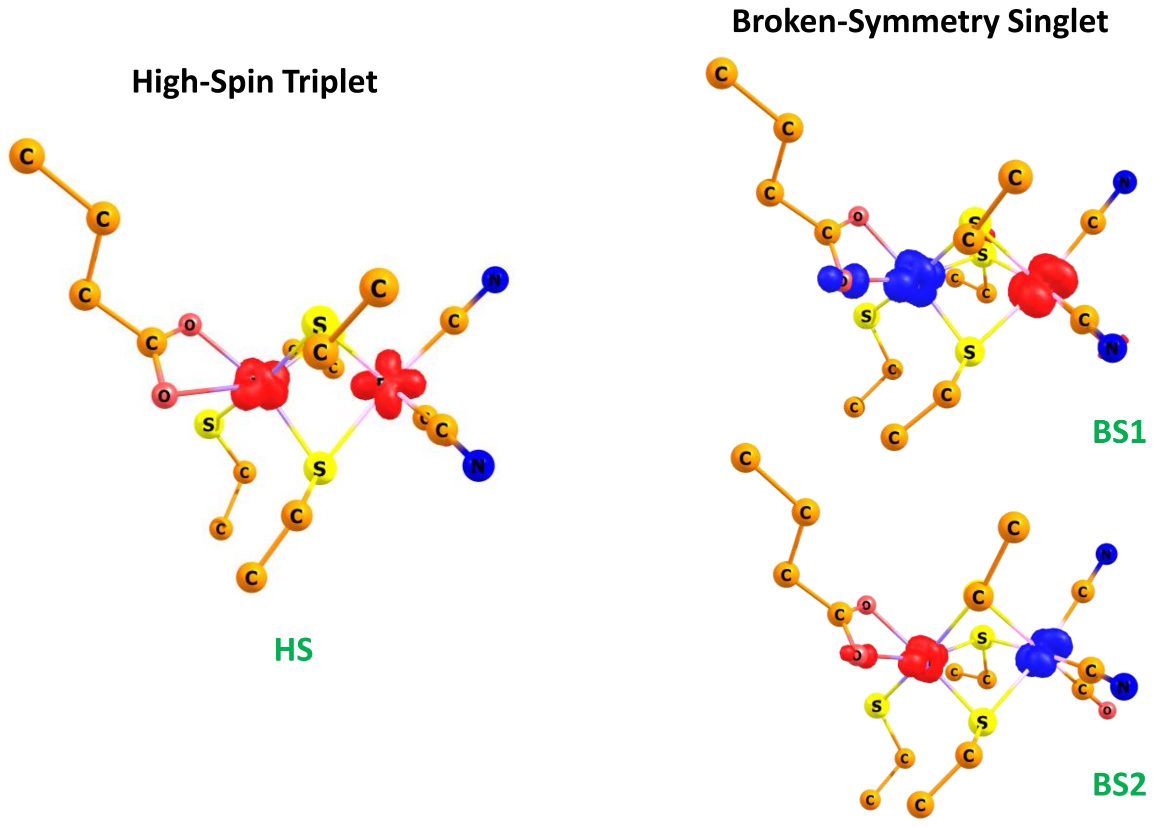

Figure S3. Distribution of unpaired electron spin densities (isocontour value 0.03 a.u.) for high-spin (HS, triplet) and broken-symmetry (BS1 and BS2, open-shell singlet) configurations (BP86/def2TZVP). BS1 and BS2 are energetically degenerate (within 0.1 kJ/mol).

Table S5: Relative energies (in kJ/mol) of high-spin configurations using various functionals with increasing number of unpaired electrons. All high-spin states are energetically higher than the triplet.

**No. of unpaired electrons**	**BP86**	**B3LYP**	**PBE0**
**2**	0.0	0.0	0.0
**4**	51.8	37.1	30.1
**6**	179.2	101.7	101.1
**8**	278.0	101.1	96.1

Table S6. Overview of Mössbauer parameters in heterobimetallic NiFe complexes and hydrogenase enzymes.

**Oxidation State Ni/Fe**	**NBO Spin densities Ni/Fe**	**^{57}Fe Mössbauer parameters (mm s^{-1}) $\delta/\Delta E_Q$**	**Ref.**
**Model Complex**			
Ni(I)/Fe(II)	0.58/0.04	n.a.	[26]
Ni(I)/Fe(II)	0.63/-0.006	n.a.	[27]
Ni(II)/Fe(I)	0.19/0.73	0.04/0.68	[28]
Ni(II)/Fe(II)	n.a.	0.25/0.24	[29]
Ni(II)/Fe(II)	n.a.	0.18/-1.52	[29]
Ni(I)/Fe(II)	0.68/-0.06	0.14/-0.73	[30]
Ni(I)/Fe(I)	n.a.	0.06/1.39	[31]
Ni(II)/Fe(II)	n.a.	0.07/0.56	[32]
**Hydrogenase**			
Ni(III)/Fe(II)	n.a.	0.45/1.2	[33]
Ni-Sia	n.a.	0.10/1.60	[34]
Ni-C	n.a.	0.07/1.69	[34]

Cartesian Coordinates of Optimized Structures

CS_BP86

Energy = -5289.476335379

Fe	-41.5716383	-23.9109354	11.2870127
C	-42.5066941	-24.1426155	12.9111260
N	-43.0010233	-24.2324586	13.9734784
C	-42.3302105	-25.4696993	10.5133295
N	-42.7859790	-26.4281314	10.0125449
C	-42.8592629	-22.9352905	10.6411269
O	-43.7308083	-22.2928346	10.2157148
Ni	-38.6325154	-23.3135413	11.4446633
C	-36.5632701	-24.2838159	12.2992597
O	-37.3043911	-23.6354425	13.1068419
O	-36.9064367	-24.3873829	11.0746201
S	-39.7662949	-25.2502906	11.9997555
S	-40.3588001	-22.1590407	12.1500864
S	-37.4133083	-21.5611337	10.7342032
S	-40.0006972	-23.6459547	9.6782689
C	-33.8099199	-27.0092279	12.7068621
H	-33.7116770	-28.0356054	12.3245802
H	-32.9448137	-26.4324473	12.3442808
H	-33.7407032	-27.0533200	13.8047833
C	-37.6064427	-18.8907150	11.4603659
H	-38.1451140	-17.9322746	11.3749774
H	-37.5475003	-19.1651385	12.5222350
H	-36.5798471	-18.7398482	11.0966897
C	-39.2058526	-21.1024191	14.4227958
H	-39.4536263	-20.0923144	14.0666099
H	-39.1600566	-21.0763379	15.5224931
H	-38.2126155	-21.3722639	14.0396355
C	-39.5824489	-27.0329499	14.0697438
H	-38.6393518	-27.4098260	13.6483212
H	-39.5800093	-27.2250552	15.1543605
H	-40.4077093	-27.6025992	13.6221761
C	-39.2860594	-21.8234834	7.6966550
H	-39.5374047	-20.9057839	7.1424629
H	-38.3007425	-21.6997620	8.1684761
H	-39.2218662	-22.6514247	6.9771124
C	-35.2869950	-24.9377775	12.7773143
H	-35.2739834	-24.9055767	13.8764160
H	-34.4476123	-24.3152214	12.4226497
C	-35.1240786	-26.3709759	12.2572074
H	-35.1905834	-26.3548288	11.1600879
H	-35.9758873	-26.9771973	12.6041182
C	-38.3214980	-19.9829648	10.6664293
H	-38.4317640	-19.6870564	9.6108747
H	-39.3300431	-20.1470482	11.0748888
C	-40.2441799	-22.1185029	13.9665630
H	-41.2637394	-21.8857756	14.3070590
H	-40.0163019	-23.1261827	14.3255121
C	-39.7502277	-25.5381599	13.8097817

CS_B3LYP

Energy = -5287.769374281

Fe	-41.6102398	-23.9290777	11.3361032
C	-42.5120381	-24.2047105	13.0018628
N	-42.9462438	-24.3096431	14.0741692
C	-42.4372018	-25.4319430	10.4639608
N	-42.9152215	-26.3247767	9.8979293
C	-42.9116571	-22.9018399	10.7360782
O	-43.7665262	-22.2503462	10.3408094
Ni	-38.6331532	-23.3443573	11.3790883
C	-36.5718719	-24.2561221	12.2151385
O	-37.3697381	-23.6062869	12.9602027
O	-36.8655354	-24.4069449	11.0002197
S	-39.7618151	-25.3066328	11.9193159
S	-40.3223603	-22.1950816	12.1845773
S	-37.4108975	-21.5629658	10.6465033
S	-40.0156848	-23.6206318	9.6333644
C	-33.8365920	-26.9049490	12.9674030
H	-33.7277583	-27.9559315	12.6912323
H	-32.9658147	-26.3667333	12.5827710
H	-33.8076213	-26.8426727	14.0585882
C	-37.5621998	-18.8992026	11.3995297
H	-38.0914897	-17.9429546	11.3304337
H	-37.4741178	-19.1714182	12.4518402
H	-36.5534938	-18.7579383	11.0060128
C	-39.1045039	-21.1122155	14.4183533
H	-39.4788428	-20.1281858	14.1296035
H	-38.9915192	-21.1279685	15.5059052
H	-38.1222517	-21.2555299	13.9682265
C	-39.7025236	-27.1588573	13.9309462
H	-38.8568580	-27.6503444	13.4458110
H	-39.6634105	-27.3759967	15.0023554
H	-40.6215857	-27.5882528	13.5307481
C	-39.3875467	-21.7637301	7.6467419
H	-39.6680555	-20.8391041	7.1339030
H	-38.3812631	-21.6541961	8.0506093
H	-39.3767869	-22.5704045	6.9121136
C	-35.3076076	-24.8442735	12.7831864
H	-35.3188428	-24.7066133	13.8657206
H	-34.4705063	-24.2609122	12.3850932
C	-35.1317905	-26.3192637	12.4135671
H	-35.1588377	-26.4126341	11.3267683
H	-35.9876684	-26.8863940	12.7905431
C	-38.3106342	-19.9807404	10.6268563
H	-38.4542045	-19.6747317	9.5874456
H	-39.2958646	-20.1341305	11.0668482
C	-40.0545292	-22.2152192	13.9806217
H	-41.0548991	-22.1189809	14.4073747
H	-39.6808327	-23.1923467	14.2663734
C	-39.6703799	-25.6503757	13.7115800

H	-38.9098019	-24.9658282	14.2235397
H	-40.7004694	-25.1718798	14.2192803
C	-40.3413839	-22.1044893	8.7557099
H	-41.3358359	-22.2694407	8.3146007
H	-40.4405715	-21.2832103	9.4781448

HS_BP86

Energy = -5289.471326791

Fe	-41.2034305	-23.8700875	11.3657127
C	-42.1651873	-24.1402968	12.9846915
N	-42.6959910	-24.2633758	14.0241058
C	-42.1871662	-25.3616476	10.6989370
N	-42.7857915	-26.2704758	10.2639406
C	-42.3953060	-22.8072315	10.6243177
O	-43.1715557	-22.1116645	10.1187657
Ni	-38.5694271	-23.1757948	11.4529118
C	-36.3454160	-24.1212362	12.3721365
O	-36.9474292	-23.6729171	13.3738004
O	-36.8012727	-24.0355746	11.1666524
S	-39.4622122	-25.2059217	12.0302114
S	-40.2243879	-22.0268085	12.3568626
S	-37.4994688	-21.3297705	10.8582258
S	-39.8021554	-23.7934102	9.5295724
C	-33.9988649	-27.2138400	12.5560299
H	-34.1957809	-28.2922364	12.4645714
H	-33.2593688	-26.9414971	11.7871576
H	-33.5337547	-27.0399531	13.5388193
C	-38.3322038	-18.7578089	11.5883381
H	-38.9225056	-17.8626679	11.3305446
H	-38.6253441	-19.0888034	12.5927455
H	-37.2708725	-18.4732943	11.6237486
C	-39.3318910	-20.9362137	14.7207595
H	-40.0207690	-20.0941182	14.5621385
H	-39.1749023	-21.0514591	15.8041816
H	-38.3652080	-20.6931342	14.2592727
C	-40.1442637	-26.9984691	13.9698450
H	-39.5567672	-27.7554641	13.4313410
H	-40.1721934	-27.2704860	15.0364622
H	-41.1718873	-27.0151658	13.5847201
C	-39.0130067	-22.0264540	7.5631380
H	-39.2457305	-21.1535519	6.9330922
H	-38.0963260	-21.8200296	8.1337237
H	-38.8229029	-22.8857186	6.9045790
C	-35.0365731	-24.8839600	12.5184980
H	-34.5989623	-24.6418383	13.4975945
H	-34.3399641	-24.5591320	11.7303423
C	-35.2811979	-26.3962800	12.3983318
H	-35.7475671	-26.5992962	11.4229593
H	-36.0154620	-26.7009506	13.1614357
C	-38.5678753	-19.8722908	10.5736975
H	-38.3539395	-19.5226617	9.5514068

H	-38.7448143	-25.2167028	14.0874371
H	-40.5245243	-25.1807394	14.1935179
C	-40.3774529	-22.0626152	8.7600968
H	-41.3848116	-22.2145756	8.3659855
H	-40.4374278	-21.2542852	9.4863597

HS_B3LYP

Energy = -5287.785515840

Fe	-41.3676669	-23.9640373	11.3218301
C	-42.2432399	-24.2557946	13.0171423
N	-42.7203383	-24.3855355	14.0653679
C	-42.3726018	-25.4688565	10.6446606
N	-42.9792273	-26.3567156	10.2145753
C	-42.6362754	-22.8609476	10.6454505
O	-43.4207331	-22.1685104	10.2035940
Ni	-38.4998925	-23.2262266	11.3948643
C	-36.3620261	-24.1022826	12.2568323
O	-37.0673516	-23.5426435	13.1283828
O	-36.7426186	-24.1599302	11.0443985
S	-39.5269190	-25.2560479	11.8745059
S	-40.2644340	-22.1027253	12.2694948
S	-37.4098464	-21.3796379	10.6493329
S	-39.9966332	-23.7120642	9.4585428
C	-33.9522486	-27.0462343	12.9927869
H	-34.0971397	-28.1290420	12.9877246
H	-33.1557373	-26.8122202	12.2812039
H	-33.6003471	-26.7608751	13.9879986
C	-37.9131258	-18.7873776	11.5666482
H	-38.5022920	-17.8688947	11.4668075
H	-37.9876469	-19.1351469	12.5968353
H	-36.8665307	-18.5439808	11.3725205
C	-39.1432746	-21.0806959	14.5613272
H	-39.6295519	-20.1255479	14.3534228
H	-39.0001137	-21.1639633	15.6422360
H	-38.1625881	-21.0866631	14.0855130
C	-40.0396092	-27.1524373	13.7818880
H	-39.4421593	-27.8597503	13.2037929
H	-40.0115355	-27.4513887	14.8336299
H	-41.0721834	-27.2124268	13.4380959
C	-39.3564534	-21.8725392	7.5081908
H	-39.6054731	-20.9488216	6.9772764
H	-38.3664698	-21.7667490	7.9523179
H	-39.3254658	-22.6870373	6.7819495
C	-35.0664207	-24.7888901	12.6261740
H	-34.7519551	-24.4372094	13.6100955
H	-34.3034408	-24.5108435	11.8947093
C	-35.2402511	-26.3124903	12.6317466
H	-35.5918707	-26.6274393	11.6471800
H	-36.0285301	-26.5790690	13.3420561
C	-38.4133873	-19.8572498	10.6035675
H	-38.3895341	-19.4869369	9.5751630

H	-39.6203693	-20.1840075	10.6035238
C	-39.8844445	-22.2319346	14.1442379
H	-40.8361301	-22.5404925	14.6008638
H	-39.1499189	-23.0369719	14.2517182
C	-39.5433968	-25.6042892	13.8131281
H	-38.5127830	-25.5376950	14.1866619
H	-40.1708512	-24.8697688	14.3247299
C	-40.1654839	-22.3140710	8.5166845
H	-41.0987777	-22.5290848	7.9757287
H	-40.3556099	-21.4621769	9.1802311

BS_BP86

Energy = -5289.476973115

Fe	-41.3871393	-24.0331268	11.2837312
C	-42.2331277	-24.3996634	12.9313028
N	-42.6749385	-24.5616083	14.0082823
C	-42.0773476	-25.5984827	10.4621700
N	-42.4926011	-26.5632478	9.9387389
C	-42.7598432	-23.1122069	10.7383061
O	-43.6851744	-22.5078119	10.3752340
Ni	-38.4949189	-23.2279239	11.3632607
C	-36.3645766	-24.1321687	12.1247187
O	-37.1121042	-23.5585315	12.9813553
O	-36.7184037	-24.1634282	10.8984449
S	-39.4718195	-25.2725327	11.8512911
S	-40.2631736	-22.2352656	12.1836368
S	-37.4267948	-21.3510653	10.7398517
S	-39.9016543	-23.5837878	9.6327189
C	-34.1454607	-27.1530173	13.0963439
H	-34.3611458	-28.2289464	13.1675847
H	-33.3252138	-27.0221797	12.3738809
H	-33.7789821	-26.8183239	14.0789549
C	-37.7621526	-18.6813368	11.3833359
H	-38.3792053	-17.7700149	11.3176109
H	-37.5761780	-18.9013232	12.4434045
H	-36.7922997	-18.4717141	10.9094294
C	-39.1707323	-21.0394307	14.4069273
H	-39.6783052	-20.1001480	14.1447742
H	-39.0099164	-21.0506991	15.4959495
H	-38.1897533	-21.0552910	13.9117861
C	-39.9005384	-27.1301501	13.7940587
H	-39.2439191	-27.8193780	13.2445247
H	-39.8881441	-27.4056132	14.8601964
H	-40.9252053	-27.2526231	13.4201888
C	-39.3670217	-21.6304399	7.7248966
H	-39.6925842	-20.7080477	7.2191754
H	-38.3807900	-21.4614925	8.1808695
H	-39.2634383	-22.4185205	6.9662365
C	-35.1118290	-24.8557051	12.5550031
H	-34.7806590	-24.4504218	13.5220312
H	-34.3238104	-24.6755988	11.8080299

H	-39.4482524	-20.1055100	10.8350731
C	-39.9782024	-22.2512403	14.0660339
H	-40.9592384	-22.3029436	14.5367026
H	-39.4563821	-23.1829009	14.2530286
C	-39.5119303	-25.7306007	13.6363736
H	-38.4790063	-25.6382523	13.9715633
H	-40.1348871	-25.0490658	14.2053149
C	-40.3898539	-22.1515185	8.5898961
H	-41.3863464	-22.2588125	8.1590279
H	-40.4208100	-21.3387610	9.3129800

BS_B3LYP

Energy = -5287.785417759

Fe	-41.3866558	-24.0165723	11.2799048
C	-42.2667892	-24.2753706	12.9817096
N	-42.7526327	-24.3864959	14.0279596
C	-42.3078385	-25.5778960	10.6234952
N	-42.8686238	-26.5028777	10.2093639
C	-42.6969197	-22.9505691	10.6387449
O	-43.5128006	-22.2741406	10.2292647
Ni	-38.4737060	-23.2169340	11.4028747
C	-36.3330056	-24.1020889	12.2508947
O	-37.0150520	-23.5522689	13.1436808
O	-36.7392766	-24.1428484	11.0431307
S	-39.5011661	-25.2477044	11.8779033
S	-40.2698110	-22.1273696	12.2207998
S	-37.3813020	-21.3693717	10.6621726
S	-39.9924675	-23.7015526	9.4745163
C	-33.9585386	-27.0729426	12.9866247
H	-34.1298343	-28.1507315	13.0354040
H	-33.1917492	-26.8906010	12.2288349
H	-33.5522814	-26.7549692	13.9507992
C	-37.8322280	-18.7726856	11.5853925
H	-38.4207130	-17.8514627	11.5092961
H	-37.8626066	-19.1175988	12.6189354
H	-36.7938386	-18.5348641	11.3454085
C	-39.1836653	-21.0594631	14.5072197
H	-39.6300441	-20.0967512	14.2507951
H	-39.0734478	-21.1071883	15.5941168
H	-38.1913494	-21.1159365	14.0597362
C	-40.0357601	-27.1348417	13.7901535
H	-39.4211774	-27.8421094	13.2302817
H	-40.0268362	-27.4245738	14.8448391
H	-41.0604719	-27.2063715	13.4255760
C	-39.3823040	-21.8262662	7.5465907
H	-39.6444436	-20.8970929	7.0319073
H	-38.3937470	-21.7142865	7.9925579
H	-39.3403706	-22.6273651	6.8061932
C	-35.0345397	-24.8039352	12.5784417
H	-34.6622274	-24.4233467	13.5311106
H	-34.3043861	-24.5743579	11.7986717

C -35.3843754 -26.3661199 12.6701753
H -35.7558762 -26.7303195 11.7010520
H -36.1994265 -26.5279182 13.3930944
C -38.4684788 -19.8559455 10.7077685
H -38.7088690 -19.6098999 9.6611439
H -39.4137968 -20.0799799 11.2240645
C -39.9910385 -22.2516795 13.9858523
H -40.9919587 -22.2924249 14.4387960
H -39.4719799 -23.1807766 14.2424925
C -39.4529271 -25.6792324 13.6336074
H -38.4297879 -25.5091631 13.9950298
H -40.1531823 -25.0237456 14.1613672
C -40.3755364 -22.0322237 8.7908862
H -41.3654451 -22.2497046 8.3623487
H -40.5164670 -21.2530796 9.5519111

CS_PBE0
Energy = -5286.466371741
Fe -41.5816592 -23.9267612 11.3485627
C -42.4931524 -24.1941179 12.9887536
N -42.9418283 -24.2969350 14.0552766
C -42.4036962 -25.4138437 10.4859085
N -42.8839992 -26.3071987 9.9234075
C -42.8820727 -22.9256273 10.7478281
O -43.7484690 -22.2937808 10.3528419
Ni -38.6341866 -23.3597384 11.4166072
C -36.6055459 -24.2551292 12.2374972
O -37.3980975 -23.6092821 12.9835465
O -36.9070046 -24.4058732 11.0284819
S -39.7687473 -25.2800810 11.9377282
S -40.3150392 -22.2223658 12.1828433
S -37.4234193 -21.5989422 10.6952641
S -39.9990086 -23.6295725 9.6939616
C -33.8090827 -26.8375020 12.8934749
H -33.6650394 -27.8733045 12.5772566
H -32.9542246 -26.2570714 12.5339424
H -33.7838687 -26.8170848 13.9869540
C -37.5024435 -18.9339932 11.3510980
H -38.0225678 -17.9724161 11.2822565
H -37.3515537 -19.1729622 12.4054716
H -36.5176818 -18.8206590 10.8915320
C -39.1507441 -21.0988726 14.3991306
H -39.5578988 -20.1277976 14.1078122
H -39.0344980 -21.1073493 15.4867620
H -38.1636255 -21.2104748 13.9465094
C -39.7919370 -27.1398236 13.9080167
H -38.9620191 -27.6565849 13.4203263
H -39.7709269 -27.3758789 14.9762797
H -40.7243812 -27.5263503 13.4929789
C -39.3497569 -21.8046003 7.7118725
H -39.6236959 -20.8852658 7.1854157

C -35.2445199 -26.3212589 12.6559130
H -35.6501962 -26.6676257 11.7029863
H -36.0037520 -26.5373732 13.4132524
C -38.3789644 -19.8429108 10.6477416
H -38.3961505 -19.4747070 9.6182832
H -39.4049315 -20.0857019 10.9224996
C -40.0462097 -22.2178274 14.0297103
H -41.0418855 -22.2156038 14.4710898
H -39.5680790 -23.1609098 14.2698345
C -39.5158573 -25.7103364 13.6427484
H -38.4897645 -25.6070985 13.9953039
H -40.1537166 -25.0298137 14.1964497
C -40.4111542 -22.1372061 8.6237586
H -41.4063853 -22.2548583 8.1927937
H -40.4538755 -21.3364479 9.3601602

CS_TPSSh
Energy = -5288.824701089
Fe -41.5472435 -23.9477912 11.3280435
C -42.4607771 -24.2216191 12.9701518
N -42.9280135 -24.3289375 14.0344683
C -42.3309576 -25.4735332 10.4867491
N -42.7968932 -26.3936201 9.9437418
C -42.8582401 -22.9604024 10.7155672
O -43.7312643 -22.3270892 10.3109291
Ni -38.6166142 -23.3432579 11.4182704
C -36.5731446 -24.2432931 12.2405569
O -37.3620015 -23.6032861 13.0062706
O -36.8898973 -24.3749593 11.0224242
S -39.7291977 -25.2857527 11.9480939
S -40.3237100 -22.2156913 12.1739637
S -37.4191411 -21.5655313 10.7072557
S -39.9830496 -23.6356985 9.6769055
C -33.8251365 -26.9074725 12.8656478
H -33.7293513 -27.9545068 12.5669085
H -32.9681970 -26.3622989 12.4579454
H -33.7577698 -26.8630101 13.9570216
C -37.5117643 -18.8820157 11.3435082
H -38.0431489 -17.9254590 11.2774099
H -37.3401300 -19.1140499 12.3962956
H -36.5377841 -18.7649862 10.8619090
C -39.1953187 -21.0692132 14.4082906
H -39.6279049 -20.1088870 14.1170161
H -39.0777314 -21.0767953 15.4964280
H -38.2077271 -21.1590459 13.9519644
C -39.8140011 -27.1353053 13.9465043
H -38.9880348 -27.6693282 13.4701553
H -39.8062418 -27.3607292 15.0180343
H -40.7507681 -27.5039070 13.5251641
C -39.3479868 -21.7756361 7.7076146
H -39.6194430 -20.8453208 7.1981284

H -38.3443477 -21.6945452 8.1225358
H -39.3365303 -22.6209017 6.9868936
C -35.3376154 -24.8311446 12.7926259
H -35.3637633 -24.7373336 13.8806565
H -34.5156285 -24.2026234 12.4303846
C -35.1142304 -26.2738876 12.3616475
H -35.1367715 -26.3197605 11.2705169
H -35.9541384 -26.8848786 12.7078284
C -38.3067554 -20.0254930 10.6672198
H -38.5122938 -19.7505730 9.6276912
H -39.2675901 -20.1529478 11.1718784
C -40.0586512 -22.2294637 13.9653825
H -41.0652909 -22.1701574 14.3890269
H -39.6507527 -23.1950787 14.2545488
C -39.7017041 -25.6371536 13.7128797
H -38.7622593 -25.2410284 14.1020081
H -40.5453097 -25.1433767 14.1953099
C -40.3417641 -22.0868674 8.8183847
H -41.3507277 -22.2359950 8.4222764
H -40.4020521 -21.2730307 9.5425316

HS_PBE0

Energy = -5286.487593844

Fe -41.3739532 -23.9238391 11.3325603
C -42.2706380 -24.1923468 12.9996391
N -42.7654998 -24.3093317 14.0409531
C -42.4169005 -25.3895856 10.6754476
N -43.0513465 -26.2600877 10.2517624
C -42.6052771 -22.8207611 10.6390795
O -43.3763773 -22.1253249 10.1842624
Ni -38.5347459 -23.2530364 11.4096599
C -36.4200255 -24.1061923 12.2464160
O -37.1325633 -23.5344252 13.1004749
O -36.7960319 -24.1916773 11.0409384
S -39.5871286 -25.2381396 11.8996102
S -40.2736050 -22.1041622 12.2736609
S -37.4477343 -21.4283061 10.6464889
S -40.0101168 -23.7325250 9.4995694
C -33.9271056 -26.9699850 12.9285041
H -34.0212716 -28.0571183 12.8681356
H -33.1156763 -26.6679460 12.2597505
H -33.6236824 -26.7175307 13.9488561
C -37.8241172 -18.8496274 11.5969899
H -38.3823137 -17.9090044 11.5271024
H -37.8719982 -19.2042965 12.6276322
H -36.7764460 -18.6432690 11.3658842
C -39.1918755 -21.0914724 14.5638240
H -39.6778147 -20.1378380 14.3441361
H -39.0673280 -21.1707219 15.6477316
H -38.2013716 -21.0953999 14.1052427
C -39.9686324 -27.1521366 13.7904899

H -38.3422584 -21.6776368 8.1206783
H -39.3431413 -22.5808582 6.9695388
C -35.2983193 -24.8418300 12.7714176
H -35.2867064 -24.7232597 13.8571923
H -34.4677027 -24.2551080 12.3628116
C -35.1396192 -26.3116593 12.3680753
H -35.2038777 -26.3821603 11.2800009
H -35.9831712 -26.8846252 12.7657726
C -38.3252631 -19.9892052 10.6777248
H -38.5441548 -19.7265490 9.6383744
H -39.2759192 -20.1237773 11.1984241
C -40.0818445 -22.2269819 13.9744270
H -41.0888843 -22.1919373 14.3963772
H -39.6426338 -23.1824534 14.2501468
C -39.6920012 -25.6282053 13.7416966
H -38.7485323 -25.2451020 14.1329309
H -40.5330454 -25.1151302 14.2056929
C -40.3453220 -22.0710328 8.8164318
H -41.3549622 -22.2146811 8.4226118
H -40.3941029 -21.2728574 9.5581627

HS_TPSSh

Energy = -5288.829887841

Fe -41.3367931 -23.8683109 11.3376044
C -42.2535351 -24.1394104 12.9986006
N -42.7591543 -24.2631107 14.0408725
C -42.4021606 -25.3193564 10.6691540
N -43.0450359 -26.1869586 10.2355360
C -42.5356433 -22.7438495 10.6300166
O -43.2960288 -22.0278828 10.1638298
Ni -38.5759063 -23.2330540 11.4144297
C -36.4423400 -24.1037590 12.2353522
O -37.1448309 -23.5379615 13.1118911
O -36.8433590 -24.1787763 11.0279240
S -39.5906114 -25.2261181 11.9239655
S -40.2633675 -22.0570286 12.3057539
S -37.4806485 -21.4324277 10.6449706
S -39.9675004 -23.7391931 9.4970360
C -33.9469802 -26.9921801 12.8977635
H -34.0622010 -28.0795483 12.8866563
H -33.1765015 -26.7283570 12.1668682
H -33.5788646 -26.7034421 13.8871308
C -37.9565805 -18.8585120 11.6209845
H -38.5087257 -17.9163614 11.5172505
H -38.0972701 -19.2333305 12.6354653
H -36.8923478 -18.6509536 11.4849917
C -39.1606095 -21.1156115 14.6370512
H -39.6603653 -20.1601411 14.4595326
H -39.0260373 -21.2370688 15.7165836
H -38.1759283 -21.0936622 14.1676875
C -39.9226250 -27.1574624 13.8229510

H	-39.2917405	-27.8034150	13.2329806
H	-39.9453622	-27.4458965	14.8442361
H	-40.9811803	-27.3096361	13.4148131
C	-39.3327430	-21.9827703	7.5127380
H	-39.5540698	-21.0676070	6.9546385
H	-38.3355537	-21.8966138	7.9484951
H	-39.3344291	-22.8207862	6.8119581
C	-35.1191634	-24.7590817	12.6333299
H	-34.8557337	-24.4416318	13.6443917
H	-34.3422138	-24.4109123	11.9458887
C	-35.2258817	-26.2783992	12.5549439
H	-35.5281677	-26.5548281	11.5417414
H	-36.0300129	-26.6155403	13.2179181
C	-38.3953742	-19.8888457	10.6508872
H	-38.4028687	-19.5103453	9.6235758
H	-39.4305325	-20.1074449	10.9217801
C	-40.0086395	-22.2609509	14.0564537
H	-40.9957870	-22.3257146	14.5175122
H	-39.4803633	-23.1923373	14.2430808
C	-39.5737280	-25.6939602	13.6521810
H	-38.5618496	-25.5053172	14.0165827
H	-40.2768073	-25.0687422	14.1980631
C	-40.3598164	-22.1931888	8.6063029
H	-41.3657246	-22.2730578	8.1876816
H	-40.3507278	-21.3603700	9.3108499

BS_PBE0

Energy = -5286.487118442

Fe	-41.3873840	-23.9425760	11.3194774
C	-42.2785851	-24.1919244	12.9941961
N	-42.7705763	-24.3035404	14.0374335
C	-42.4156988	-25.4229008	10.6781924
N	-43.0459424	-26.2993979	10.2604668
C	-42.6229395	-22.8405449	10.6371140
O	-43.3948322	-22.1397801	10.1914561
Ni	-38.5214157	-23.2437880	11.4128404
C	-36.4118183	-24.1070770	12.2599404
O	-37.1140433	-23.5395919	13.1230157
O	-36.7977651	-24.1851935	11.0551460
S	-39.5831282	-25.2270118	11.9030572
S	-40.2730746	-22.1090663	12.2605529
S	-37.4260560	-21.4238705	10.6576225
S	-40.0061057	-23.7238130	9.5142337
C	-33.9231475	-26.9804675	12.9204825
H	-34.0216788	-28.0672612	12.8615526
H	-33.1134459	-26.6817718	12.2475215
H	-33.6145336	-26.7286353	13.9394642
C	-37.8125094	-18.8424141	11.5970834
H	-38.3780409	-17.9059869	11.5261956
H	-37.8530842	-19.1933735	12.6294482
H	-36.7677553	-18.6294585	11.3603423

H	-39.2024577	-27.7752592	13.2811930
H	-39.9073737	-27.4471336	14.8786682
H	-40.9186624	-27.3595051	13.4257532
C	-39.2634239	-22.0191940	7.4714596
H	-39.4834555	-21.1162289	6.8919502
H	-38.2755795	-21.9178461	7.9242872
H	-39.2516652	-22.8723003	6.7888308
C	-35.1296827	-24.7635629	12.5904267
H	-34.8174447	-24.4160212	13.5774985
H	-34.3789118	-24.4571198	11.8561032
C	-35.2650847	-26.2924385	12.5752744
H	-35.6276454	-26.6008583	11.5915929
H	-36.0302944	-26.5908324	13.2992414
C	-38.4432822	-19.8845485	10.6040947
H	-38.3564133	-19.4891904	9.5872647
H	-39.4962988	-20.1101305	10.7804162
C	-39.9764416	-22.2787279	14.0931852
H	-40.9611671	-22.3663366	14.5544259
H	-39.4382492	-23.2116363	14.2318704
C	-39.5904353	-25.6746198	13.6935818
H	-38.5940032	-25.4413811	14.0727337
H	-40.3352719	-25.0775794	14.2142592
C	-40.3128632	-22.2120943	8.5573574
H	-41.3123325	-22.3152419	8.1295731
H	-40.3196551	-21.3698648	9.2489315

BS_TPSSh

Energy = -5288.830043286

Fe	-41.4073184	-24.0096359	11.2318713
C	-42.3197482	-24.2348774	12.9041470
N	-42.8372159	-24.3285007	13.9437018
C	-42.2404425	-25.6112565	10.6045822
N	-42.7469578	-26.5804467	10.2049618
C	-42.7302264	-22.9999690	10.6054891
O	-43.5851567	-22.3481391	10.2115801
Ni	-38.5146941	-23.2157592	11.4229601
C	-36.3949170	-24.1045901	12.2287609
O	-37.0764101	-23.5421739	13.1211896
O	-36.8105554	-24.1439615	11.0207214
S	-39.5345945	-25.2099742	11.9075195
S	-40.3080731	-22.1446792	12.1658016
S	-37.4219928	-21.3940274	10.6852737
S	-39.9769513	-23.6811955	9.5302022
C	-34.0484143	-27.0978319	12.9448855
H	-34.2329356	-28.1740304	13.0034469
H	-33.3012081	-26.9272652	12.1638681
H	-33.6125037	-26.7794550	13.8969001
C	-37.6654824	-18.7747488	11.5737542
H	-38.2278762	-17.8331324	11.5563095
H	-37.5663765	-19.1029073	12.6098770
H	-36.6614800	-18.5804850	11.1879697

C	-39.2028013	-21.0827461	14.5489255	C	-39.2397763	-21.0892312	14.4545670
H	-39.6826266	-20.1272058	14.3230531	H	-39.6145102	-20.1057532	14.1607877
H	-39.0797040	-21.1561584	15.6330504	H	-39.1611701	-21.1155079	15.5460674
H	-38.2122998	-21.0953511	14.0911494	H	-38.2433041	-21.2263713	14.0313060
C	-39.9683896	-27.1482803	13.7856198	C	-39.7314127	-27.1337917	13.8392306
H	-39.2864199	-27.7948416	13.2288204	H	-38.9130681	-27.6710596	13.3539761
H	-39.9482872	-27.4457159	14.8385842	H	-39.7412474	-27.3980417	14.9018858
H	-40.9784989	-27.3094115	13.4050703	H	-40.6710081	-27.4643088	13.3933754
C	-39.3352500	-21.9747718	7.5248836	C	-39.3637575	-21.8265567	7.5846170
H	-39.5607154	-21.0619438	6.9641904	H	-39.6226464	-20.8960061	7.0686412
H	-38.3399911	-21.8818645	7.9636871	H	-38.3717892	-21.7219646	8.0282279
H	-39.3298601	-22.8135743	6.8253136	H	-39.3363010	-22.6321953	6.8471826
C	-35.1082305	-24.7652560	12.6288285	C	-35.1036309	-24.8166400	12.5547090
H	-34.8315493	-24.4484793	13.6363409	H	-34.7203661	-24.4328601	13.5027369
H	-34.3383778	-24.4202987	11.9319442	H	-34.3765658	-24.6037621	11.7660859
C	-35.2209247	-26.2843022	12.5527811	C	-35.3350770	-26.3323416	12.6452037
H	-35.5297375	-26.5619536	11.5418123	H	-35.7693558	-26.6762828	11.7029862
H	-36.0226008	-26.6176133	13.2210271	H	-36.0767235	-26.5335851	13.4246333
C	-38.3819743	-19.8892598	10.6581804	C	-38.3728169	-19.8412498	10.7426033
H	-38.3961483	-19.5142583	9.6296357	H	-38.5118741	-19.4931798	9.7141708
H	-39.4146829	-20.1120625	10.9349775	H	-39.3621540	-20.0477504	11.1565099
C	-40.0269945	-22.2501764	14.0479306	C	-40.1698575	-22.1980757	13.9814076
H	-41.0174946	-22.3012122	14.5025550	H	-41.1825453	-22.1046247	14.3749343
H	-39.5091403	-23.1842347	14.2485316	H	-39.7824652	-23.1726173	14.2643496
C	-39.5789609	-25.6877889	13.6540480	C	-39.5698769	-25.6251395	13.6862389
H	-38.5698611	-25.4955759	14.0240982	H	-38.6272777	-25.2692176	14.1047623
H	-40.2875889	-25.0667479	14.1978292	H	-40.4041819	-25.1097019	14.1571950
C	-40.3649690	-22.1892381	8.6152202	C	-40.3880241	-22.1220419	8.6711060
H	-41.3685194	-22.2779025	8.1938125	H	-41.3910985	-22.2386927	8.2557400
H	-40.3638877	-21.3558100	9.3190904	H	-40.4146221	-21.3258796	9.4168120

References:

1. Furche, F.; Ahlrichs, R.; Hättig, C.; Klopper, W.; Sierka, M.; Weigend, F., Turbomole. *Wiley Interdiscip. Rev. Comput. Mol. Sci.* **2014,** *4* (2), 91-100.
2. Turbomole v. 7.5.1 2019, A development of University of Karlsruhe and Forschungszentrum Karlsruhe GmbH, 1989-2007, since 2007 available from TURBOMOLE GmbH (www.turbomole.com).
3. Treutler, O.; Ahlrichs, R., Efficient molecular numerical integration schemes. *J. Chem. Phys.* **1995,** *102* (1), 346-354.
4. Grimme, S.; Antony, J.; Ehrlich, S.; Krieg, H., A consistent and accurate ab initio parametrization of density functional dispersion correction (DFT-D) for the 94 elements H-Pu. *J. Chem. Phys.* **2010,** *132* (15), 154104.
5. Grimme, S.; Ehrlich, S.; Goerigk, L., Effect of the damping function in dispersion corrected density functional theory. *J. Comput. Chem.* **2011,** *32* (7), 1456-1465.
6. Becke, A. D., Density-functional exchange-energy approximation with correct asymptotic behavior. *Phys. Rev. A* **1988,** *38* (6), 3098.
7. Perdew, J. P., Density-functional approximation for the correlation energy of the inhomogeneous electron gas. *Physical Review B* **1986,** *33* (12), 8822-8824.
8. Perdew, J. P., Erratum: Density-functional approximation for the correlation energy of the inhomogeneous electron gas. *Physical Review B* **1986,** *34* (10), 7406-7406.
9. Becke, A. D., Density-functional thermochemistry. III. The role of exact exchange. *J. Chem. Phys.* **1993,** *98* (7), 5648-5652.
10. Lee, C.; Yang, W.; Parr, R. G., Development of the Colle-Salvetti correlation-energy formula into a functional of the electron density. *Phys. Rev. B* **1988,** *37* (2), 785-789.
11. Ernzerhof, M.; Scuseria, G. E., Assessment of the Perdew–Burke–Ernzerhof exchange-correlation functional. *The Journal of Chemical Physics* **1999,** *110* (11), 5029-5036.
12. Adamo, C.; Barone, V., Toward reliable density functional methods without adjustable parameters: The PBE0 model. *The Journal of Chemical Physics* **1999,** *110* (13), 6158-6170.

13. Staroverov, V. N.; Scuseria, G. E.; Tao, J.; Perdew, J. P., Comparative assessment of a new nonempirical density functional: Molecules and hydrogen-bonded complexes. *J. Chem. Phys.* **2003,** *119* (23), 12129-12137.
14. Staroverov, V. N.; Scuseria, G. E.; Tao, J.; Perdew, J. P., Erratum: "Comparative assessment of a new nonempirical density functional: Molecules and hydrogen-bonded complexes" [J. Chem. Phys. 119, 12129 (2003)]. *J. Chem. Phys.* **2004,** *121* (22), 11507-11507.
15. Schäfer, A.; Horn, H.; Ahlrichs, R., Fully optimized contracted Gaussian basis sets for atoms Li to Kr. *J. Chem. Phys.* **1992,** *97* (4), 2571-2577.
16. Noodleman, L.; Davidson, E. R., Ligand spin polarization and antiferromagnetic coupling in transition metal dimers. *Chem. Phys.* **1986,** *109* (1), 131-143.
17. Noodleman, L.; Baerends, E. J., Electronic structure, magnetic properties, ESR, and optical spectra for 2-iron ferredoxin models by LCAO-X. alpha. valence bond theory. *J. Am. Chem. Soc.* **1984,** *106* (8), 2316-2327.
18. Noodleman, L.; Peng, C.; Case, D.; Mouesca, J.-M., Orbital interactions, electron delocalization and spin coupling in iron-sulfur clusters. *Coord. Chem. Rev.* **1995,** *144*, 199-244.
19. Noodleman, L., Valence bond description of antiferromagnetic coupling in transition metal dimers. *J. Chem. Phys.* **1981,** *74* (10), 5737-5743.
20. Shomura, Y.; Taketa, M.; Nakashima, H.; Tai, H.; Nakagawa, H.; Ikeda, Y.; Ishii, M.; Igarashi, Y.; Nishihara, H.; Yoon, K.-S., Structural basis of the redox switches in the NAD+-reducing soluble [NiFe]-hydrogenase. *Science* **2017,** *357* (6354), 928-932.
21. Kulka-Peschke, C. J.; Schulz, A.-C.; Lorent, C.; Rippers, Y.; Wahlefeld, S.; Preissler, J.; Schulz, C.; Wiemann, C.; Bernitzky, C. C.; Karafoulidi-Retsou, C., Reversible Glutamate Coordination to High-Valent Nickel Protects the Active Site of a [NiFe] Hydrogenase from Oxygen. *J. Am. Chem. Soc.* **2022,** *144* (37), 17022-17032.
22. Neese, F., Software update: the ORCA program system, version 4.0. *Wiley Interdiscip. Rev. Comput. Mol. Sci.* **2018,** *8* (1), e1327.
23. Neese, F., The ORCA program system. *Wiley Interdiscip. Rev. Comput. Mol. Sci.* **2012,** *2* (1), 73-78.
24. Yamaguchi, K.; Fukui, H.; Fueno, T., Molecular orbital (MO) theory for magnetically interacting organic compounds. Ab-initio MO calculations of the effective exchange integrals for cyclophane-type carbene dimers. *Chem. Lett.* **1986,** *15* (4), 625-628.
25. Yamaguchi, K.; Takahara, Y.; Fueno, T.; Houk, K., Extended Hartree-Fock (EHF) theory of chemical reactions. *Theor. Chim. Acta* **1988,** *73* (5), 337-364.
26. Zhu, W.; Marr, A. C.; Wang, Q.; Neese, F.; Spencer, D. J. E.; Blake, A. J.; Cooke, P. A.; Wilson, C.; Schröder, M., Modulation of the electronic structure and the Ni–Fe distance in heterobimetallic models for the active site in [NiFe]hydrogenase. *Proceedings of the National Academy of Sciences* **2005,** *102* (51), 18280-18285.
27. Barton, B. E.; Whaley, C. M.; Rauchfuss, T. B.; Gray, D. L., Nickel−Iron Dithiolato Hydrides Relevant to the [NiFe]-Hydrogenase Active Site. *Journal of the American Chemical Society* **2009,** *131* (20), 6942-6943.
28. Schilter, D.; Nilges, M. J.; Chakrabarti, M.; Lindahl, P. A.; Rauchfuss, T. B.; Stein, M., Mixed-valence nickel-iron dithiolate models of the [NiFe]-hydrogenase active site. *Inorganic Chemistry* **2012,** *51* (4), 2338-48.
29. Kochem, A.; Bill, E.; Neese, F.; van Gastel, M., Mössbauer and computational investigation of a functional [NiFe] hydrogenase model complex. *Chemical Communications* **2015,** *51* (11), 2099-2102.
30. Chambers, G. M.; Huynh, M. T.; Li, Y.; Hammes-Schiffer, S.; Rauchfuss, T. B.; Reijerse, E.; Lubitz, W., Models of the Ni-L and Ni-SIa States of the [NiFe]-Hydrogenase Active Site. *Inorganic chemistry* **2016,** *55* (2), 419-431.
31. Weber, K.; Krämer, T.; Shafaat, H. S.; Weyhermüller, T.; Bill, E.; van Gastel, M.; Neese, F.; Lubitz, W., A Functional [NiFe]-Hydrogenase Model Compound That Undergoes Biologically Relevant Reversible Thiolate Protonation. *Journal of the American Chemical Society* **2012,** *134* (51), 20745-20755.
32. C. Davies, S.; J. Evans, D.; L. Hughes, D.; Longhurst, S.; Roger Sanders, J., Synthesis and structure of a thiolate-bridged nickel–iron complex: towards a mimic of the active site of NiFe-hydrogenase. *Chemical Communications* **1999,** (19), 1935-1936.
33. Surerus, K. K.; Chen, M.; van der Zwaan, J. W.; Rusnak, F. M.; Kolk, M.; Duin, E. C.; Albracht, S. P. J.; Muenck, E., Further Characterization of the Spin Coupling Observed in Oxidized Hydrogenase from Chromatium vinosum. A Moessbauer and Multifrequency EPR Study. *Biochemistry* **1994,** *33* (16), 4980-4993.
34. Roncaroli, F.; Bill, E.; Friedrich, B.; Lenz, O.; Lubitz, W.; Pandelia, M.-E., Cofactor composition and function of a H2-sensing regulatory hydrogenase as revealed by Mössbauer and EPR spectroscopy. *Chemical Science* **2015,** *6* (8), 4495-4507.
35. Römelt, M.; Ye, S.; Neese, F. Calibration of Modern Density Functional Theory Methods for the Prediction of 57Fe Mo¨ssbauer Isomer Shifts: Meta-GGA and Double-Hybrid Functionals. *Inorg. Chem.*, **2009**, *48* (3), 784-785.
36. Preissler, J.; Wahlefeld, S.; Lorent, C.; Teutloff, C.; Horch, M.; Lauterbach, L.; Cramer, S. P.; Zebger, I.; Lenz, O. Enzymatic and Spectroscopic Properties of a Thermostable [NiFe]-hydrogenase Performing H2-Driven NAD+-Reduction in the Presence of O2. *Biochim. Biophys. Acta - Bioenerg.* **2018**, *1859* (1), 8–18.
